# Sociocultural considerations in food addiction and emotional eating: comparative evidence from college students in the United States and South Korea

**DOI:** 10.3389/fpubh.2026.1890961

**Published:** 2026-09-09

**Authors:** Aidan Mahoney, Susan Sugarman, Kaylee Hough, Attila Szabo, Nicole Avena

**Affiliations:** 1 Department of Psychology, Princeton University, Princeton, NJ, United States; 2 Interdisciplinary Affective Science Lab, Department of Psychology, Northeastern University, Boston, MA, United States; 3 Department of Neuroscience, Ichan School of Medicine at Mount Sinai, New York, NY, United States; 4 Faculty of Health and Sport Sciences, Széchenyi István University, Győr, Hungary

**Keywords:** cross-cultural comparison, emotional eating, food addiction, South Korea, ultra-processed foods, undergraduate students

## Abstract

Food addiction and emotional eating are two eating-related constructs that have been linked to obesity and poor mental health, but the extent to which they reliably co-occur across different cultural contexts is less clear. Given that sociocultural norms and food environments differ between countries, this study examined whether food addiction and emotional eating are associated in undergraduate samples from the United States (*n* = 110) and South Korea (*n* = 141), and whether scores differ across key demographic subgroups within each country. Using a cross-sectional design, food addiction was assessed with the Yale Food Addiction Scale 2.0 (YFAS 2.0), and emotional eating was assessed with the Emotional Eating Scale–II (EES-II). Consistent with the study’s central hypothesis, food addiction and emotional eating were strongly and positively correlated in both samples (South Korea *r* = 0.74; United States *r* = 0.53; both *p* < 0.001), and this association was significantly stronger in the South Korean sample (Fisher’s *z* = 2.82, *p* = 0.005). In a hierarchical regression, emotional eating remained a robust predictor of food addiction symptoms after adjusting for country, age, gender, and sexual orientation (Δ*R*^2^ = 0.25, *p* < 0.001). Additionally, cisgender women reported higher emotional eating than cisgender men overall (*p* = 0.007), while gender differences in food addiction symptoms were not statistically significant (*p* = 0.069). Overall, these findings indicate pronounced cross-cultural differences in both food addiction symptoms and emotional eating, suggesting that sociocultural factors may shape how these eating-related behaviors are expressed, highlighting the importance of culturally informed strategies to address emotional regulation in increasingly obesogenic food environments worldwide.

## 1 Introduction

The obesity epidemic in the United States (U.S.) represents a critical public health challenge, with recent data revealing that 40.3% of American adults meet clinical criteria for obesity (1). A significant driver is the widespread availability and consumption of ultra-processed foods, defined as “industrially formulated edible substances derived from natural food that are highly profitable, convenient, and hyper-palatable” (2). Ultra-processed food consumption is not just a concern in the United States; it is a growing issue worldwide. One randomized clinical trial in Japan demonstrated that participants consuming ultra-processed foods for 1 week ingested an average of 813 additional calories per day compared to those consuming minimally processed foods (3).

Literature suggests that food addiction may contribute to the emergence and continued reinforcement of maladaptive eating patterns. Studies have demonstrated that intermittent, excessive sugar intake produces neurological imbalances similar to opioid dependence (4), with documented parallels between substance use disorders and overeating, including altered dopamine signaling (5), craving patterns (6–8), and evidence that highly processed foods are more likely to be implicated in addictive-like eating behaviors (9). Critically, epidemiological data estimates suggest that approximately 20% of the general population may meet diagnostic criteria for food addiction, highlighting the public health significance of this emerging construct (10).

South Korea and the United States provide a compelling cross-cultural comparison of eating-related constructs for several reasons. They represent collectivist and individualist cultures, respectively, which have differing norms for emotion regulation strategies and are therefore a key factor in emotional eating behaviors (11, 12). For example, one study by Guidinger et al. (13) found that emotional dysregulation was linked to loss-of-control eating in Asian/Asian American men, an eating behavior which has been associated with psychosocial distress, depression, weight gain, and obesity (14, 15). Additionally, South Korea has experienced a rapid nutritional shift, with Western-style hyperpalatable foods becoming widespread amid a traditionally low-fat, low-sugar diet (16). Hence, it provides a unique context for studying how increased exposure to processed foods interacts with culturally ingrained, healthier eating habits (9).

Furthermore, both the prevalence of eating disorders and obesity rates in South Korea have risen dramatically in recent years, highlighting an increasing public health issue (17, 18). Although the Yale Food Addiction Scale 2.0 (YFAS 2.0) has been validated in Korean samples (19), no known research has directly compared food addiction and emotional eating between South Korean and U.S. samples, a gap highlighted by recent calls for cross-cultural studies of food addiction that takes into account cultural differences regarding eating behaviors and emotional regulation (20).

Given the relevance of social identity to eating-related risk, the present study also examined gender identity and sexual orientation as exploratory demographic variables. Prior research with college students suggests that eating-related pathology may vary across gender identity and sexual orientation, with sexual and gender minority students reporting higher eating-disorder symptoms than heterosexual and cisgender peers (21). These disparities may reflect differences in emotion-regulation demands, exposure to social stress, stigma, and coping processes, all of which are theoretically relevant to emotional eating and addictive-like eating behaviors. However, because subgroup sizes for gender-diverse and sexual minority participants were expected to be limited in the present cross-cultural sample, these analyses were treated as exploratory rather than confirmatory.

The main goal of this study was to examine the relationships between emotional eating and food addiction among college students in South Korea and the United States, with the hypothesis that the two measures would reliably and positively co-occur for participants. Despite growing evidence that ultra-processed foods activate neural reward pathways associated with addiction, little research has investigated how these relationships manifest across cultures with distinct food traditions and eating practices. Understanding cross-cultural patterns in emotional eating and food addiction is critical for developing culturally informed interventions that address the mental and physical health challenges facing college students in an increasingly ultra-processed food environment.

## 2 Materials and methods

### 2.1 Participants

Data were collected from undergraduate students at two country sites: Princeton, New Jersey in the United States and multiple universities in Seoul, South Korea. In the U.S., 900 randomly selected Princeton University undergraduate students were contacted via email to participate in an online survey. Recruitment materials included an overview of the study purpose and incentive information. The final U.S. sample consisted of 110 participants who completed the survey over a three-week recruitment period in the spring of 2024.

In South Korea, participants were recruited over a two-week period in the summer of 2024 by distributing flyers with quick response (QR) codes at three major universities in Seoul: Seoul National University, Korea University, and Yonsei University. The QR code linked directly to the survey, and additional participants from other Seoul-area universities were recruited through snowball sampling. The final South Korean sample consisted of 141 participants who completed the survey.

All participants reviewed an informed consent page detailing survey duration, content, confidentiality protections, and a warning regarding potential triggering content for individuals with disordered eating histories. Participants provided electronic consent before proceeding and received a full debriefing upon completion. This study was approved by the Princeton University Institutional Review Board.

### 2.2 Measures

The survey consisted of 50 items: 25 questions from the Yale Food Addiction Scale 2.0 [YFAS 2.0; Gearhardt et al. (22)] and 25 questions from the Emotional Eating Scale-II [EES-II; Ruiz et al. (23)]. Demographic variables assessed included gender identity, age, sexual orientation, and athletic participation. The U.S. sample additionally assessed race/ethnicity. The U.S. sample completed the survey in English, while the South Korean sample completed a Korean translation prepared by a native Korean speaker with proficiency in both Korean and English. The translator was instructed to prioritize conceptual equivalence over literal translation, and particular attention was given to adapting item wording that referenced Western-specific concepts. While the translated instruments were reviewed for clarity and face validity, the present study did not employ a formal forward-backward translation procedure or independent expert panel review, representing a limitation of the current study design.

The YFAS 2.0 is a validated self-report questionnaire assessing behaviors associated with food addiction (22). It has demonstrated robust psychometric properties as a self-report questionnaire for identifying and quantifying food addiction behaviors across diverse cultural and linguistic contexts (24–26). The 25-item scale uses a 5-point frequency response format (0 = Never, 4 = Always), evaluating symptoms including loss of control, persistent cravings, and continued consumption despite negative consequences (22). The YFAS 2.0 yields two scoring metrics: a symptom count reflecting the number of addiction-like criteria endorsed, and a diagnostic threshold indicating whether clinically significant impairment or distress accompanies three or more symptoms. The present study utilized the symptom count scoring method, in which higher total scores indicate greater food addiction symptom severity, without requiring a clinical diagnosis for the participant.

The EES-II is a validated instrument evaluating the frequency with which individuals eat in response to various emotional states (23), based off empirical findings from multiple international samples, including both the United States and South Korea. Cross-cultural validation studies provide robust evidence supporting the instrument’s factorial structure, internal consistency reliability, measurement invariance across populations, and construct validity for assessing emotional eating behaviors in psychological and public health research (23). The 25-item scale uses a 5-point Likert response format ranging from “No Desire to Eat” (0) to “An Overwhelming Urge to Eat” (4). The EES-II assesses eating responses to four emotional domains: anger, anxiety, depression, and positive mood. Higher total scores indicate greater emotional eating tendencies. Consistent with established guidelines, the EES-II was employed as a dimensional measure rather than for categorical diagnosis to provide a best fit of comparison with the YFAS 2.0 (27).

### 2.3 Procedure

Recruitment and survey access procedures differed by site. U.S. participants accessed the survey via an embedded email link, while South Korean participants accessed it via QR codes on recruitment flyers distributed on university campuses. After providing informed consent, all participants completed the YFAS 2.0, followed by the EES-II, and then demographic questions. South Korean participants also reported their current university enrollment status, and those not enrolled were excluded from the final dataset. On the penultimate page, South Korean participants provided their university email address for enrollment verification. Incentive distribution for both sites was sent to the first 25 respondents via email. A debriefing statement about the purpose of the study was presented upon survey submission at both sites.

Data were collected via Qualtrics Experience Management (XM) software. To maintain confidentiality, each participant was assigned a unique anonymous identifier, ensuring that the researcher could not link email addresses to survey responses. Incentive distribution for the first 25 completers at each site was handled separately. At the U.S. site, alphabetized participant email lists were sent directly to the researcher from the Office of Undergraduate Research (OUR) after the data collection was finalized. The first 25 respondents each received a $10 gift card to Small World Coffee. At the South Korean site, participant university enrollment was verified by matching self-reported university affiliation with the provided email domain, and incentive delivery was confirmed to valid email addresses after the two-week data collection period. The first 25 participants in the Korean sample were given a $10 Amazon gift card.

### 2.4 Sample size calculation and data analysis

Data were analyzed in Statistical Package for the Social Sciences (SPSS) using multivariate analysis of variance (MANOVA) with YFAS 2.0 and EES-II total scores as dependent variables. An *a priori* power analysis in G*Power 3.1.9.7 (28) indicated a minimum required sample of *N* = 152; the final sample (*N* = 251; U.S. *n* = 110, South Korea *n* = 141) exceeded this threshold. Cross-tabulations were used to assess cell sizes, and gender identity was excluded from the primary model due to sparse/imbalanced cells; the primary analysis therefore tested country × sexual orientation effects using Pillai’s Trace (given a significant Box’s M), followed by univariate analysis of variance (ANOVA) tests with effect size (partial Eta squared [pη^2^]) reported for significant multivariate effects. A secondary country × cisgender (female vs. male) MANOVA was conducted on complete cases to examine gender differences in survey responses.

To directly test the study’s central hypothesis, Pearson correlations between YFAS 2.0 and EES-II total scores were computed for the full sample and separately within each country, with 95% confidence intervals (CIs) obtained via Fisher’s *r*-to-*z* transformation; the two country-specific correlations were compared using Fisher’s *z* test for independent correlations. To evaluate whether emotional eating predicted food addiction symptoms independently of demographic factors, a two-step hierarchical linear regression was estimated with YFAS 2.0 as the outcome: Step 1 entered country, age, cisgender identity, and sexual orientation, and Step 2 added EES-II scores; a country × emotional-eating interaction term was then tested to determine whether the emotional-eating–food-addiction slope differed across countries. For multivariate and univariate effects, 90% CIs for partial η^2^ were derived from the noncentral *F* distribution (the 90% interval is conventional for partial η^2^ because the statistic is bounded at zero). Because the country × sexual orientation and country × cisgender models were fitted on overlapping subsamples drawn from the same 251 participants, they are reported as complementary analyses rather than independent replications, and the two univariate country effects within each model were evaluated against a Bonferroni-adjusted *α* of 0.025. Both dependent variables were complete for all 251 participants; the reduced *N* in the cisgender analysis reflects a definitional restriction to cisgender women and men rather than item-level missingness, and this exclusion mechanism was examined by comparing included and excluded participants on both outcomes. The YFAS 2.0 and EES-II have shown good-to-excellent internal consistency in prior Korean and U.S./international validation studies (19, 22, 23, 59); because the analytic dataset retained scale totals rather than item-level responses, sample-specific reliability coefficients could not be recomputed and this is noted as a limitation. Analyses were conducted in SPSS and in Python (SciPy, statsmodels).

## 3 Results

### 3.1 Sample characteristics

A total of 251 participants completed the survey and were included for data analysis: 110 from the U.S. sample and 141 from the South Korean sample. Response rates for demographic items varied across samples and categories.

The U.S. sample comprised 81 cisgender women (73.6%), 25 cisgender men (22.7%), 5 non-binary individuals (4.5%), and 1 genderqueer individual (0.1%), with a few participants selecting multiple gender identities. Ages ranged from 18 to 22 years old. Race/ethnicity was diverse, with the largest groups being 51 participants identifying as White (46.4%) and 45 participants identifying as Asian (40.9%). 79 participants identified as heterosexual (71.8%), with 30 other participants stating a sexual orientation on the LGBTQ+ spectrum (27.3%).

The South Korean sample comprised 64 cisgender women (45.4%), 56 cisgender men (39.7%), 10 non-binary individuals (7.1%), 2 genderqueer individuals (1.4%), 2 transgender women (1.4%), and 1 transgender man (0.7%). 6 participants did not state their gender identity (4.3%). Age ranged from 18 to 23 years old. 95 participants identified as heterosexual (67.4%), with 41 participants stating a sexual orientation on the LGBTQ+ spectrum (29.1%). 5 participants (3.5%) did not state their sexual orientation. Race/ethnicity data were not taken due to the homogeneous makeup of South Korean universities.

### 3.2 Cross-cultural comparison

An *a priori* power analysis was conducted to determine the minimum sample size for a MANOVA examining the effects of country (2 levels), gender (3 levels), and sexual orientation (2 levels) on two dependent variables (YFAS 2.0 and EES-II). The analysis was specified for MANOVA special effects and interactions (*F* test), with f^2^(V) = 0.0625 (Pillai’s V = 0.118), *α* = 0.05, power = 0.95, 12 groups (2 × 3 × 2 factorial design), two predictors (corresponding to the highest-df effects), and two response variables. The required sample size was 152 participants (actual power = 0.951; critical *F* = 2.404; *λ* = 19.00; numerator df = 4; denominator df = 280).

Box’s M test of equality of covariance matrices was statistically significant (M = 28.63, *F*(9, 108727.67) = 3.12, *p* < 0.001), indicating a violation of the homogeneity of covariance matrices assumption. Given this violation, Pillai’s Trace was selected as the multivariate test statistic because it is the most robust to this violation (29). Levene’s test of equality of error variances was nonsignificant for both YFAS 2.0 scores (*F*(3, 241) = 1.98, *p* = 0.118) and EES-II scores (*F*(3, 241) = 2.38, *p* = 0.070), which indicated that the assumption of homogeneity of variances was met for both dependent measures.

A 2 (country: South Korea vs. United States) × 2 (sexual orientation: heterosexual vs. non-heterosexual) between-participants MANOVA was calculated for two dependent variables: YFAS 2.0 scores and EES. Using Pillai’s Trace, there was a statistically significant multivariate main effect for country, V = 0.336, *F*(2, 240) = 60.84, *p* < 0.001, effect size [pη^2^] = 0.336. The multivariate main effect for sexual orientation was not significant, V = 0.010, *F*(2, 240) = 1.24, *p* = 0.291, pη^2^ = 0.010. The Country × Sexual Orientation interaction was also not significant, V = 0.002, *F*(2, 240) = 0.23, *p* = 0.797, pη^2^ = 0.002.

Cross tabulation for the feasibility of the planned MANOVA with two dependent measures (YFAS 2.0 and EES-II).

As shown in Table 1, “gender identity” was excluded from the MANOVA due to a highly unbalanced distribution of participants across gender identity categories within country grouping. As seen in Panel A, while the cisgender subgroups were adequately represented (*n* = 117 and *n* = 103), the remaining categories had substantially smaller cell sizes (*n* = 15, *n* = 9, *n* = 6, and *n* = 1). Thus, including gender identity as an additional factor would have introduced numerous sparse cells into the factorial design. Such small cell sizes compromise the stability of variance–covariance matrix estimates, reducing statistical power. They also undermine the reliability of multivariate test statistics (29). For these reasons, gender identity was excluded from the MANOVA (Table 2).

**Table 1:** Sample characteristics by country.

Panel A. Gender identity	Cisgender	Non-cisgender	Other	Total
South Korea	117	15	9	141
United States	103	6	1	110
Total	220	21	10	251

Panel B. Sexual orientation	Heterosexual	Non-heterosexual	Total
South Korea	95	41	136
United States	79	30	109
Total	174	71	245
Missing cases	0 (Gender)	6 (Sexual orientation)	251 total sample

Cell sizes are presented for gender identity and sexual orientation by country. Gender identity was coded into three categories: cisgender, non-cisgender, and other. Sexual orientation was coded into two categories: heterosexual and non-heterosexual. Six participants were missing data for sexual orientation.

**Table 2:** Cell sizes for the planned MANOVA by country and cisgender identity.

Panel A. Cisgender identity	Female	Male	Other	Total
South Korea	66	51	24	141
United States	79	24	7	110
Total	145	75	31	251

This table presents the cross-tabulation used to evaluate the feasibility of the planned MANOVA with YFAS 2.0 and EES-II as dependent variables. Cisgender identity was categorized as female, male, or other based on self-reported gender identity. Participants in gender categories with insufficient sample sizes for comparison were grouped into the “Other” category.

**Figure 1:**
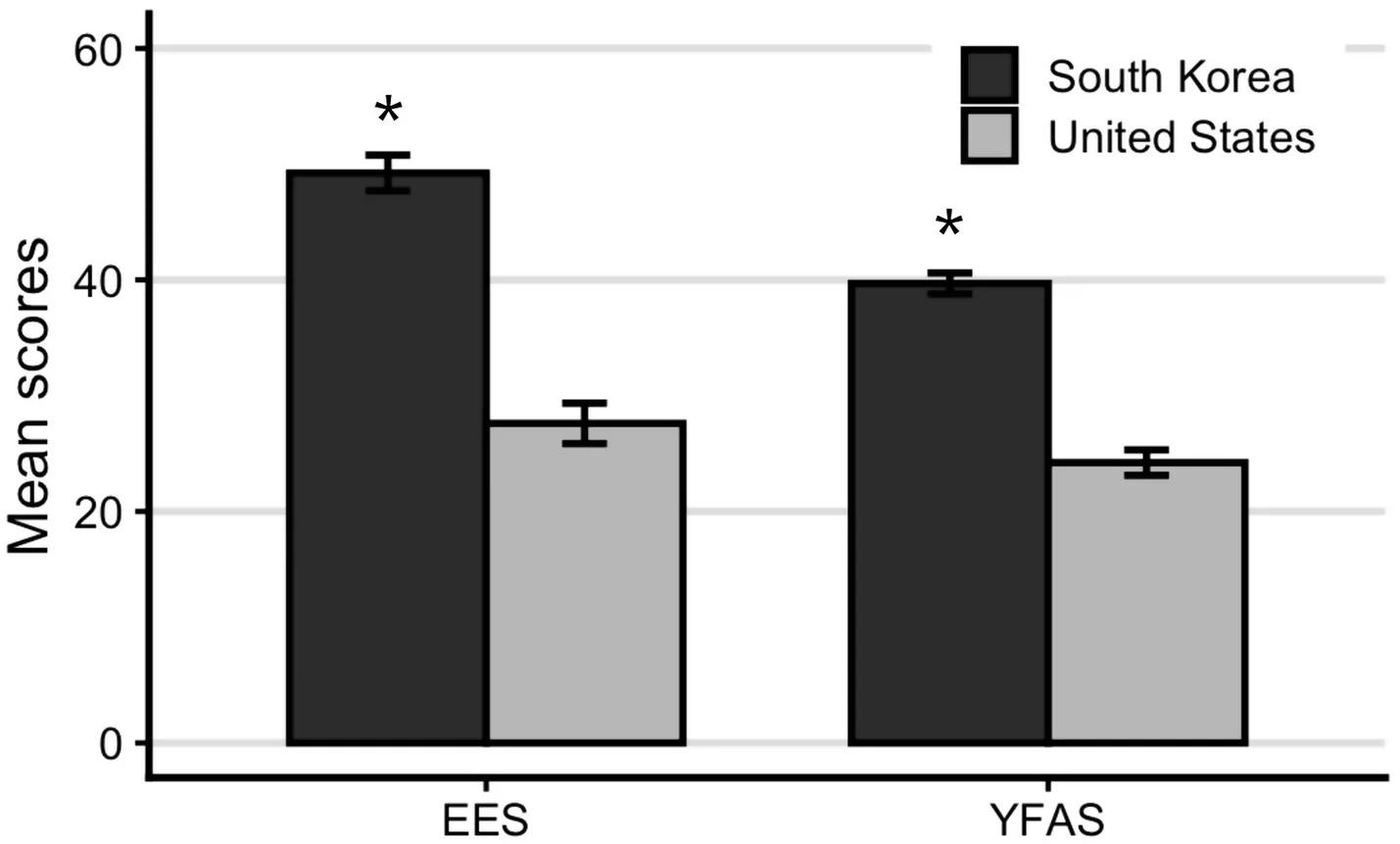
Mean EES-II and YFAS 2.0 scores by country. South Korean participants scored significantly higher than United States participants on both the Emotional Eating Scale-II (EES-II) and the Yale Food Addiction Scale 2.0 (YFAS 2.0). Error bars represent 95% confidence intervals. *p < 0.001.

Given the significant multivariate effect of country, univariate between-subjects ANOVAs were examined. Country had a significant effect on YFAS 2.0 scores, *F*(1, 241) = 116.68, *p* < 0.001, pη^2^ = 0.326, with Korean participants (M = 40.27, SD = 9.69) scoring significantly higher than U.S. participants (M = 24.34, SD = 11.51). The Country category also had a significant effect on EES-II scores, *F*(1, 241) = 75.75, *p* < 0.001, pη^2^ = 0.239, with Korean participants (M = 49.38, SD = 18.11) again scoring significantly higher than U.S. participants (M = 27.50, SD = 18.36). Neither sexual orientation nor the Country × Sexual Orientation interaction reached significance for either dependent variable (all ps > 0.14). These results are illustrated in Figure 1. Ninety-percent confidence intervals for the country effect sizes were [0.25, 0.40] for YFAS 2.0 symptom counts and [0.17, 0.31] for EES-II scores (Table 3).

**Table 3:** Descriptive statistics for YFAS 2.0 and EES-II scores by country and cisgender identity.

Panel A. YFAS 2.0 Scores	Female	Male
South Korea	40.70 (8.97)	39.53 (11.60)
United States	25.14 (11.87)	20.38 (10.60)

Panel B. EES-II Scores	Female	Male
South Korea	50.33 (17.43)	47.10 (21.03)
United States	29.92 (18.00)	18.08 (17.06)

Means and standard deviations are presented for YFAS 2.0 and EES-II scores by country and cisgender identity. Values are shown as mean (SD). Analyses were restricted to cisgender female and male participants with complete data (N = 220); 31 participants were excluded because they reported another gender identity category. Excluded participants did not differ significantly from included participants on YFAS 2.0 or EES-II scores.

### 3.3 Association between food addiction and emotional eating

The study’s central hypothesis—that food addiction and emotional eating positively co-occur—was tested directly. Across the full sample, YFAS 2.0 and EES-II total scores were strongly and positively correlated, *r*(249) = 0.74, *p* < 0.001, 95% CI [0.68, 0.79]. The association was significant and positive within each country and was descriptively stronger in the South Korean sample (*r* = 0.744, 95% CI [0.66, 0.81]) than in the U.S. sample (*r* = 0.534, 95% CI [0.39, 0.66]). A Fisher’s *z* test for independent correlations indicated that this difference in the zero-order correlations was statistically significant, *z* = 2.82, *p* = 0.005, suggesting that addictive-like and emotional eating may be more tightly coupled in the Korean context. Correlations and CIs are summarized in Table 4.

**Table 4:** Correlations between YFAS 2.0 and EES-II scores by sample.

Sample	*n*	*r* (YFAS 2.0 × EES-II)	95% CI	*p*
South Korea	141	0.744	[0.660, 0.810]	<0.001
United States	110	0.534	[0.385, 0.656]	<0.001
Full sample	251	0.740	[0.678, 0.793]	<0.001

Pearson correlations between YFAS 2.0 and EES-II scores are shown for the South Korean sample, the United States sample, and the full sample. Confidence intervals were calculated using Fisher’s r-to-z transformation. Both measures were complete for all participants, so no cases were excluded from these correlations.

To test whether emotional eating predicted food addiction symptoms independently of demographic factors, a two-step hierarchical linear regression was estimated on complete cases (*N* = 214) with YFAS 2.0 as the outcome. Step 1, comprising country, age, cisgender identity, and sexual orientation, accounted for a significant portion of variance, *R*^2^ = 0.38, *F*(4, 209) = 31.91, *p* < 0.001. Adding EES-II in Step 2 produced a large and significant increment, Δ*R*^2^ = 0.25, Δ*F*(1, 208) = 138.24, *p* < 0.001, with the full model explaining 63% of the variance in food addiction symptoms, *R*^2^ = 0.63. Emotional eating was a strong unique predictor, *b* = 0.375, 95% CI [0.312, 0.437], *β* = 0.60, *t*(208) = 11.76, *p* < 0.001, and country remained significant (Korean participants higher), *b* = −8.20, 95% CI [−11.15, −5.25], *p* < 0.001, whereas age, cisgender identity, and sexual orientation were not significant (all *p* > 0.44). Coefficients are reported in Table 5.

**Table 5:** Hierarchical linear regression predicting YFAS 2.0 symptom-count scores.

Predictor	*b*	95% CI	*β*	*p*
Step 1 (*R*^2^ = 0.38)				
Country (US vs. Korea)	−17.80	[−21.0, −14.6]	−0.65	<0.001
Step 2 (*R*^2^ = 0.63; Δ*R*^2^ = 0.25)				
Country (US vs. Korea)	−8.20	[−11.1, −5.2]	−0.31	<0.001
Age	−0.10	[−0.88, 0.69]	−0.01	0.808
Cisgender (male vs. female)	−0.13	[−2.62, 2.36]	−0.00	0.918
Sexual orientation (non-het.)	1.02	[−1.61, 3.66]	0.03	0.444
Emotional eating (EES-II)	0.375	[0.312, 0.437]	0.60	<0.001

Hierarchical regression results are presented for predictors of YFAS 2.0 symptom-count scores. Step 1 included country as a predictor, and Step 2 added age, cisgender identity, sexual orientation, and emotional eating. Country was coded 0 = South Korea and 1 = United States; cisgender identity was coded 0 = female and 1 = male; sexual orientation was coded 0 = heterosexual and 1 = non-heterosexual. Continuous predictors were mean-centered, and β represents the standardized coefficient.

Finally, a country × emotional-eating interaction term was added to the regression to test whether the emotional-eating–food-addiction slope differed across countries. This interaction was not statistically significant, *b* = −0.07, *p* = 0.30, indicating that, once between-country differences in mean levels and score dispersion were taken into account, the *within-person* coupling of emotional eating and food addiction did not differ reliably between the two countries. The significant Fisher’s *z* for the zero-order correlations should therefore be interpreted with caution, as it partly reflects country differences in variance rather than a difference in the underlying association (see Discussion).

### 3.4 Gender differences

Because this analysis draws on the same participant pool as the preceding country × sexual orientation model, the two sets of results are not statistically independent; the country effect reported here should be understood as the same effect re-estimated in an overlapping subsample rather than an independent replication. To limit Type I error inflation from testing two dependent variables within each model, univariate country effects were evaluated against a Bonferroni-adjusted *α* of 0.025, and all country effects remained significant at this threshold. To evaluate whether YFAS 2.0 and EES-II scores differed as a function of country and cisgender identity, we examined cross tabulations to assess cell sizes for a planned 2 (country: South Korea vs. United States) × 2 (gender: female vs. male) MANOVA with two dependent variables (YFAS 2.0 and EES-II).

Using Pillai’s Trace, there was a statistically significant multivariate main effect of country, V = 0.360, *F*(2, 215) = 60.54, *p* < 0.001, pη^2^ = 0.360. There was also a statistically significant multivariate main effect of cisgender, V = 0.033, *F*(2, 215) = 3.72, *p* = 0.026, partial η^2^ = 0.033. The Country × Cisgender interaction was not significant, V = 0.011, *F*(2, 215) = 1.22, *p* = 0.297, partial η^2^ = 0.011.

**Figure 2:**
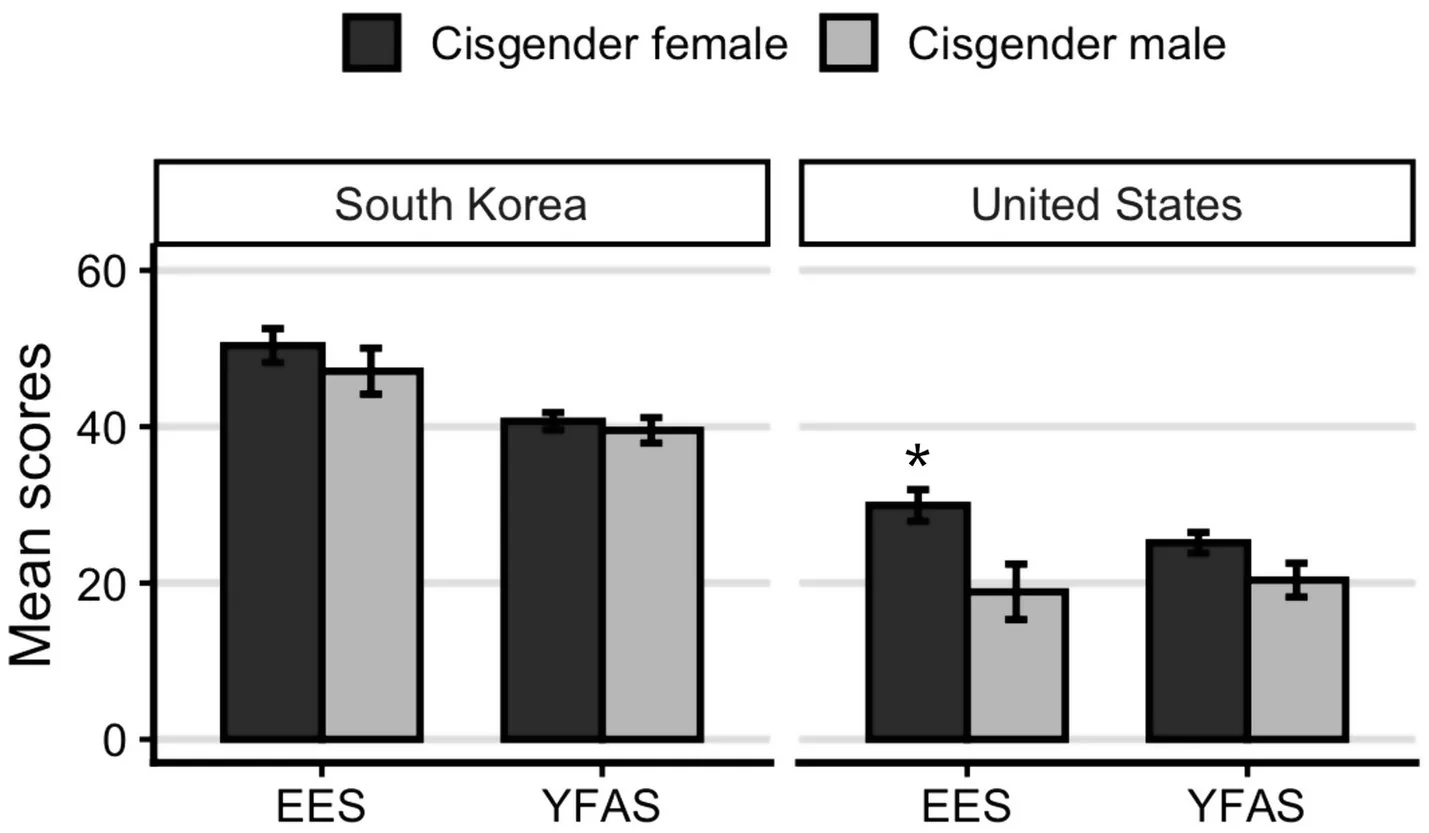
Mean EES-II and YFAS 2.0 scores by country and gender, shown separately for South Korea (left) and the United States (right). Among US participants, cisgender females scored significantly higher than cisgender males on the EES-II; no significant gender differences emerged in the South Korean sample. Error bars represent 95% confidence intervals. *p < 0.001.

Given the significant multivariate effects, univariate between-subjects ANOVAs were examined for each dependent variable. Country significantly predicted both outcomes, such that participants in Korea scored higher than participants in the United States on both YFAS 2.0, *F*(1, 216) = 114.43, *p* < 0.001, partial η^2^ = 0.346 (South Korea: M = 40.19, SD = 10.17; United States: M = 24.03, SD = 11.71), and EES-II, *F*(1, 216) = 80.22, *p* < 0.001, partial η^2^ = 0.271 (South Korea: M = 48.92, SD = 19.06; United States: M = 27.17, SD = 18.41). Cisgender differences were significant for EES-II but not for YFAS 2.0: the cisgender effect did not reach significance for YFAS 2.0, *F*(1, 216) = 3.34, *p* = 0.069, partial η^2^ = 0.015, whereas cisgender women scored higher than cisgender men on EES-II, *F*(1, 216) = 7.46, *p* = 0.007, partial η^2^ = 0.033 (women: M = 39.21, SD = 20.41; men: M = 37.81, SD = 23.98), collapsed across countries. The Country × Cisgender interaction was not significant for either YFAS 2.0 (*p* = 0.269) or EES-II (*p* = 0.120), indicating that country differences in YFAS 2.0 and EES-II did not significantly vary by cisgender group. Overall, results indicate robust cross-cultural differences, with Korean participants scoring higher on both YFAS 2.0 and EES-II, and cisgender differences emerging for emotional eating (EES-II) but not for food addiction symptoms (YFAS 2.0), in the absence of a country-by-cisgender interaction. These results are illustrated in Figure 2. Ninety-percent confidence intervals for the country effect were [0.26, 0.42] (YFAS 2.0) and [0.19, 0.35] (EES-II); the cisgender difference in EES-II had a 90% CI of [0.005, 0.081].

## 4 Discussion

### 4.1 Overview of findings

The present study investigated the sociocultural mechanisms underlying ultra-processed food consumption during emotional distress. The hypothesis that emotional eating and food addiction symptoms would positively co-occur was supported: across the historically divergent food environments of the United States and South Korea, emotional eating correlated strongly and positively with food addiction symptoms, *r* = 0.74, and this association held after adjusting for country and demographic covariates. Because the present design is cross-sectional and based on self-report, these data establish co-occurrence but cannot confirm the neurobiological or causal mechanisms discussed below; the reward-circuitry and evolutionary accounts are offered as theoretical interpretations rather than as processes tested in this study.

An evolutionary perspective may help explain this cross-cultural consistency between food addiction and emotional eating correlations. Neuroscientific evidence indicates that sugar content in ultra-processed foods reinforces emotional eating behaviors through habitual effects on neural reward systems (5). Regardless of geographic location or cultural background, individuals might remain susceptible to sugar’s biological effects on the brain (30). In ancestral environments, sugar was scarce, obtained only through natural sources such as fruits or honey (31). Consuming energy-dense foods conferred survival advantages by enabling caloric storage as adipose tissue for future energy needs during scarcity (32). Individuals consuming sugary foods demonstrated enhanced survival in uncertain environments, leading to evolutionary selection for sugar preference as protection against famine (33); therefore, this could be a reason as to why participants across the different cultures examined here demonstrated a correlation between scores on each scale.

Emotional eating and food addiction possibly interconnect through multiple neural mechanisms. The tendency of “emotion eaters” to repeatedly turn to comfort foods during stress to achieve sugar’s hedonic effects resembles dependence patterns in drug addiction (5). Simultaneously, emotional eating habits condition the brain to require escalating sugar quantities to achieve desired pleasurable responses in a way analogous to tolerance in substance abuse (5). Despite dramatic evolution in modern life and technology, the human genome and neural circuitry have changed minimally over the past 10,000 years (34), suggesting that brain architecture remains adapted to encourage increased eating during sugar abundance as preparation for potential scarcity (35). Because emotional eaters preferentially consume high-sugar ultra-processed foods during stress, it follows that these individuals are likely at greater risk to demonstrate elevated food addiction vulnerability (36). These neurobiological and evolutionary frameworks are advanced to contextualize the observed cross-cultural consistency, but they cannot be tested with the present cross-sectional, self-report data and should be regarded as hypotheses for future experimental and longitudinal work rather than as demonstrated mechanisms.

### 4.2 Cross-cultural comparison

Because most food addiction research has been conducted in Western populations, findings from the United States may not generalize to Eastern countries, where dietary patterns and meal practices have historically differed from those typical in the U.S. Consistent with this concern, country emerged as a robust predictor of both outcomes in the present study: South Korean students scored significantly higher than U.S. students on both the YFAS 2.0 and on the EES-II. In addition, the association between food addiction symptoms and emotional eating was strong in both samples but significantly larger in South Korea than in the U.S. (Korea: *r* = 0.744; U.S.: *r* = 0.534; Fisher’s *z* = 2.82, *p* = 0.005), suggesting that emotional eating and addictive-like eating may be more tightly coupled in the Korean cultural context.

The much stronger correlation between YFAS 2.0 and EES-II scores in the Korean sample than in the U.S. sample suggests that the overlap between addictive-like eating and emotional eating may be more pronounced in the Korean cultural context. The correlations align with previous research documenting a strong positive relationship between food addiction and emotional eating tendencies (22, 37), but the cultural differences in the strength of this link have received little empirical focus. One possible explanation is that emotional regulation strategies vary across collectivist and individualist cultures (12), which may influence how negative emotions affect eating behavior. In collectivist societies like South Korea, emotional suppression is more culturally accepted (11), and food might play a more central role as a coping mechanism, possibly strengthening the connection between emotional stress and addictive eating. Cultural differences in dietary environments, such as the rising availability of highly processed Western-style foods in South Korea, may also interact with existing emotional eating tendencies to enhance addictive-like responses (38).

Additionally, prevailing societal attitudes toward mental illness and professional help-seeking behaviors may influence how Koreans navigate such diet-related mental health challenges. Persistent mental illness stigma within Korean society frequently inhibits engagement with professional mental health services, leading to increased reliance on self-directed regulation strategies (i.e., emotional eating) (39). Although public health initiatives are currently being designed to promote psychological well-being through targeted dietary interventions (40), the continued expansion of ultra-processed food availability within the Korean food environment may systematically undermine efforts to promote healthful dietary choices (41). The development of culturally sensitive approaches to health promotion represents an essential priority, given the complex intersections between dietary patterns and mental health outcomes across cultural contexts. A comprehensive understanding of the interplay among traditional dietary practices, modern environmental influences, and underlying neurobiological mechanisms is necessary to develop effective intervention strategies to address food addiction and emotional eating behaviors while advancing global mental and physical health outcomes.

It is important to note that the cross-national differences observed between South Korean and U.S. participants should be interpreted in light of several unmeasured covariates, including body mass index, socioeconomic status, dietary patterns, ultra-processed food intake, perceived stress, and physical activity. Without taking these factors into account, the extent to which the observed group differences can be attributed to cultural factors alone is limited, and future research should incorporate these measures to better isolate the effects of culture from potential confounds.

In summary, while cultural differences alone cannot fully explain emotional eating behaviors and food addiction prevalence, unique psychological, sociocultural, and environmental factors in Seoul provide compelling frameworks for understanding why university students there scored higher on these measures than U.S. counterparts. Future studies should explore the specific pathways through which culture influences the relationship between food addiction and emotional eating.

### 4.3 Gender

Results from both the U.S. and Korean samples align with prior findings that women demonstrate greater susceptibility to emotional eating than men, while food addiction symptoms do not show the same consistent gender pattern. Gender differences in disordered eating behaviors are well-established (42); however, no obvious biological mechanisms fully explain these disparities. Instead, differences may reflect socialization regarding emotional expression and coping.

One potential mechanism involves gender differences in emotional responding. In one functional magnetic resonance imaging (fMRI) study, men exhibited greater amygdala deactivation when viewing negatively valenced images than women, which was associated with reduced emotional responding to aversive stimuli (43). If men experience attenuated emotional reactivity to negative affect, this may reduce the likelihood that they use food as a strategy for emotion regulation. Consistent with this view, women have reported employing more emotion regulation behaviors than men (44) and demonstrate greater vulnerability to uncontrolled emotional eating in response to stress (45).

College women may be particularly influenced by stress-related eating patterns. Under stressful conditions, female college students have demonstrated increased appetite and elevated preference for sweet foods (e.g., candy and ice cream) and mixed dishes (e.g., burgers and pizza) (46). These comfort foods often exemplify ultra-processed products whose high sugar and fat content can activate neural reinforcement pathways, supporting cycles in which emotional eating is maintained, and therefore, potentially strengthening the link between negative affect and eating behavior over time.

Whether biological differences in emotional reactivity or gender socialization explain these findings, the present results, importantly, suggest that this gender-linked pattern generalizes beyond a single cultural context: Cisgender women showed higher EES-II scores than cisgender men in both the United States and South Korea and this difference in emotional eating scores was statistically significant in the U.S. sample. This gender difference is consistent with the broader literature framing emotional eating as a stress- and affect-regulation behavior that may be particularly shaped by social norms and emotion-coping strategies. While substantially larger samples with balanced gender distributions are needed to further substantiate and refine these conclusions, the current results support the interpretation that gender differences are more robust for emotional eating than for food addiction symptoms, and that this pattern appears in both countries in the present dataset.

### 4.4 Sexual orientation

Although gender produced significant emotional eating differences among U.S. students, sexual orientation was not significantly associated with YFAS 2.0 or EES-II outcomes in either the U.S. or Korean samples. In the U.S. sample, interpretation is further limited by the small number of participants identifying as non-heterosexual (*n* = 30), which reduces statistical power and makes estimates less stable. In the cross-cultural model, a 2 (country: South Korea vs. United States) × 2 (sexual orientation: heterosexual vs. non-heterosexual) MANOVA indicated a robust main effect of country on YFAS 2.0 and EES-II scores, but no significant multivariate main effect of sexual orientation, as well as no Country × Sexual Orientation interaction. This suggests that sexual orientation did not explain additional variance in emotional eating or food addiction scores beyond the sizable differences between the Korean and U.S. groups. Accordingly, while some group means differences may appear descriptively elevated among sexual minority participants in certain subsamples, these patterns should be interpreted cautiously as non-significant trends rather than evidence of a reliable effect in either country.

This null finding does not rule out meaningful links between sexual minority status and disordered eating risk; rather, it indicates that such associations were not detectable here, potentially due to limited subgroup sizes and the broad collapsing of identity categories (e.g., “non-heterosexual”). Prior research suggests that stigma, discrimination, concealment stress, and reduced social support can contribute to chronic stress and poorer mental health outcomes among sexual and gender minority (SGM) individuals, which in turn may elevate vulnerability to maladaptive coping behaviors such as emotional eating (47–49). Studies have documented higher rates of eating disorder symptoms and related psychopathology among lesbian, gay, bisexual, and transgender (LGBT) adolescents and adults compared to heterosexual and cisgender peers (21, 50–53). However, evidence in East Asian contexts remains comparatively limited, and cultural factors (e.g., differing norms around disclosure and family/community expectations) may shape both stress exposure and help-seeking in ways that are not well captured by the present measures (54). Future work with larger, more-balanced samples of participants with different sexual orientations is needed to clarify whether sexual orientation relates to emotional eating and food addiction severity within each country, and whether any cross-cultural differences emerge.

### 4.5 Limitations

When interpreting the results from both samples, it is important to note that the recruitment channels varied from country to country. In the United States, participants were recruited from a single university (Princeton) and were able to opt-in directly through a random email invitation sent only to 900 undergraduate students. In contrast, South Korean participants were recruited from three universities via flyer QR codes (Yonsei University, Korea University, and Seoul National University), before snowball sampling resulted in participants from a few other universities also opting-in. This introduces additional confounding variables (such as differing socioeconomic status of the average student) to this study apart from differences in country alone; limiting the reliability of our cross-cultural conclusions. However, the undergraduate demographic composition at top universities in Korea’s SKY universities (Seoul National, Korea, Yonsei) overlap greatly, such that we can cautiously posit that the samples at these universities do not differ substantially from one another, and therefore, did not introduce significant differences into our data from the South Korean sample (55).

Additionally, race/ethnicity data were not collected for the South Korean sample due to the assumed homogeneous makeup of South Korean universities. This decision was made due to demographic data from Korean universities which suggest that, at most, only 10.8% (253,400) of its total undergraduate population (2,339,937) is international (56). Further, 34.5% of those students are from China (72,020), 26.8% are from Vietnam (56,003), 5.9% are from Mongolia (12,317), and 5.8% are from Uzbekistan (12,025), which are all countries that fall under the “East” vs. “West” classification (57, 60). Europe and North America only account for 5.1% (10,681) and 2.0% (4.217), respectfully (60). However, it is a methodological flaw to omit standard demographic questions based on an assumption. Therefore, we suggest that future cross-cultural work collects standardized demographic data to ensure reliability of findings.

Several limitations further qualify these conclusions. The cross-sectional, self-report design precludes causal or mechanistic inference, so the reward-circuitry and evolutionary accounts above are interpretive rather than tested. The country × sexual orientation and country × cisgender models were fitted on overlapping subsamples from the same 251 participants and thus do not constitute independent replications of the country effect. Finally, because the analytic dataset retained only scale totals, sample-specific internal-consistency coefficients for the YFAS 2.0 and EES-II could not be computed here and are instead drawn from prior validation studies; future work should report within-sample reliability.

One final limitation concerns the cross-cultural comparability of the measures used in this study. Because the primary aim involves comparing psychological constructs — food addiction (YFAS 2.0) and emotional eating (EES-II) — across English-speaking and Korean-speaking populations, whether these instruments assess the same underlying constructs equivalently across cultures is critical. Although prior validation studies support the translated versions (19), the present study did not formally test measurement invariance. This is particularly important given well-documented cross-cultural differences in response styles; for example, individuals from collectivist cultures may demonstrate acquiescence bias or midpoint responding, while those from individualist cultures may exhibit more extreme responding (58). Such systematic differences in how participants interpret and respond to items could attenuate observed group differences. Consequently, score differences between cultural groups should be interpreted with caution, as they may partly reflect differential item functioning rather than genuine differences in the constructs of interest. Future research employing multi-group confirmatory factor analysis or item response theory frameworks would help establish whether these instruments operate equivalently across populations and strengthen the validity of cross-cultural comparisons.

## 5 Conclusion

The present research examined the relationship between food addiction symptoms and emotional eating, two closely related constructs in neuroscience and psychology that share overlapping risk factors and may reflect interconnected pathways of reward sensitivity and emotion regulation. Across both university samples in the United States and South Korea, YFAS 2.0 and EES-II scores were strongly, positively correlated, indicating that greater prevalence of addictive-like eating symptoms reliably co-occurred with higher levels of emotional eating across cultural contexts.

Beyond this shared association, a significant result was a robust cross-cultural difference, with South Korean participants scoring significantly higher than U.S. participants on both the YFAS 2.0 and EES-II scales, suggesting greater overall severity or reporting of both addictive-like eating patterns and emotionally driven eating in the Korean sample. These country differences likely reflect a convergence of factors (e.g., dietary environment, social eating norms, and stress exposure) that shape eating behavior and emotion regulation in daily life.

Expanded analyses also clarified how a participant’s gender identity relates to these outcomes. Although the sexual orientation of participants showed no significant differences regardless of country, cisgender women reported higher EES-II scores than cisgender men in the U.S. and when collapsed across both countries. However, gender differences were not statistically significant for YFAS 2.0 scores in either country. The absence of a country-by-gender interaction indicates that this gender-linked elevation in emotional eating among women was consistent in both cultural contexts, suggesting that gendered patterns in emotion-related eating may be relatively stable across settings even when overall country-level means differ substantially. This divergence highlights the importance of treating these constructs (food addiction and emotional eating) as related but non-identical phenotypes, with partially distinct social and psychological determinants.

While the online methodology, incentivized participation, and uneven subgroup sizes impose limitations on inference and generalizability, the overall pattern of results emphasizes that neurobiological vulnerability, psychological coping processes, and cultural context jointly shape eating behavior. Future research should prioritize larger, more diverse samples (including balanced gender/sexuality representation) and culturally tailored intervention approaches to reduce risk for emotional and addictive-like eating among college students globally.

## Data Availability

The raw data supporting the conclusions of this article will be made available by the authors, without undue reservation.
